# ORBiT: Oak Ridge biosurveillance toolkit for public health dynamics

**DOI:** 10.1186/1471-2105-16-S17-S4

**Published:** 2015-12-07

**Authors:** Arvind Ramanathan, Laura L Pullum, Tanner C Hobson, Chad A Steed, Shannon P Quinn, Chakra S Chennubhotla, Silvia Valkova

**Affiliations:** 1 Computational Science and Engineering Division, Oak Ridge National Laboratory, One Bethel Valley Road MS6085, Oak Ridge, TN, 37830, USA; 2 Health Data Sciences Institute, Oak Ridge National Laboratory, One Bethel Valley Road MS6085, Oak Ridge, TN, 37830, USA; 3 Department of Computational & Systems Biology, 3500 Fifth Avenue, Pittsburgh, PA, 15260, USA; 4 IMS Government Solutions, One Plymouth Place, Plymouth Meeting, PA, 15260, USA

**Keywords:** Public health surveillance, non-negative matrix factorization, electronic healthcare reimbursement, H1N1 2009 Pandemic

## Abstract

**Background:**

The digitization of health-related information through electronic health records (EHR) and electronic healthcare reimbursement claims and the continued growth of self-reported health information through social media provides both tremendous opportunities and challenges in developing effective biosurveillance tools. With novel emerging infectious diseases being reported across different parts of the world, there is a need to build systems that can track, monitor and report such events in a timely manner. Further, it is also important to identify susceptible geographic regions and populations where emerging diseases may have a significant impact.

**Methods:**

In this paper, we present an overview of Oak Ridge Biosurveillance Toolkit (ORBiT), which we have developed specifically to address data analytic challenges in the realm of public health surveillance. In particular, ORBiT provides an extensible environment to pull together diverse, large-scale datasets and analyze them to identify spatial and temporal patterns for various biosurveillance-related tasks.

**Results:**

We demonstrate the utility of ORBiT in automatically extracting a small number of spatial and temporal patterns during the 2009-2010 pandemic H1N1 flu season using claims data. These patterns provide quantitative insights into the dynamics of how the pandemic flu spread across different parts of the country. We discovered that the claims data exhibits multi-scale patterns from which we could identify a small number of states in the United States (US) that act as "bridge regions" contributing to one or more specific influenza spread patterns. Similar to previous studies, the patterns show that the south-eastern regions of the US were widely affected by the H1N1 flu pandemic. Several of these south-eastern states act as bridge regions, which connect the north-east and central US in terms of flu occurrences.

**Conclusions:**

These quantitative insights show how the claims data combined with novel analytical techniques can provide important information to decision makers when an epidemic spreads throughout the country. Taken together ORBiT provides a scalable and extensible platform for public health surveillance.

## Background

Public health surveillance is the continuous, systematic collection, analysis and interpretation of health-related data for planning, implementing and evaluating public health practice. It can serve as an effective vehicle for monitoring epidemiology of various health problems, including infectious (e.g., flu, West Nile Virus, Ebola, etc.) and chronic (e.g., diabetes, cancer, etc.) health conditions, documenting the impact of interventions and/or tracking progress of specific health goals, and serve as an early warning system for impending public health emergencies [1]. As emerging and re-emerging pathogens, such as the recent Ebola virus outbreaks in West Africa [2] and the Middle Eastern Respiratory Syndrome (MERS) outbreaks [3], become more prevalent, developing effective public health surveillance systems is a priority for ensuring national security. Additionally, with the continued increase in the number of asthma, diabetes and other chronic disease conditions, there is an immediate need to develop tools that can aid decision makers (e.g., public health officials, physicians, epidemiologists and policy/law-makers) with critical information that can eventually translate into effective health policies. With an estimated 50-60 million patients diagnosed every year and continued growth of medical expenses-related to these conditions, the combined effect of these diseases is an extraordinary socioeconomic burden, which can only be overcome by developing effective public health surveillance systems.

### Public health surveillance is a big data problem

At the core of public health surveillance is the availability of health-related data, which can be broadly classified into two classes: (1) direct sources, which include health records such as data from clinical and emergency visits, poison control centers, laboratory results, hospitals, etc. and (2) indirect sources, which include health relevant information from school attendance/closure reports, sales data (for over the counter medications, prescription records, etc.), news feeds and social media. Taken together, these different datasets can exceed several petabytes of data that have to be integrated and analyzed to obtain even basic insights into how diseases spread within geographically separated populations.

With the digitization of health-related information and web-based platforms that promote self-reporting (through Twitter, Facebook and other social media sites), there has been an exponential growth of data available for public health surveillance. Current platforms for biosurveillance make use of event-based, unstructured data such as news feed aggregators and other publicly available data to monitor for emerging infectious disease spread within geographically distributed populations. Examples of such systems include the BioSense 2.0 program [4], GPHIN (Global Public Health Information Network) [5], PHIN (Public Health Information Network) [6], ProMED-Mail [7], HealthMap [8], Google Flu Trends, Bio-Caster, EpiSPIDER [9], EARS (Early Aberration Reporting System), BCON (biosurveillance Common Operating Network), PHESS (Indiana Public Health Emergency Surveillance System), LAHVA (Linked Animal-Human Health Visual Analytics), ESSENCE (Electronic Surveillance System for Early Notification of Community-based Epidemics) [10], RODS (Real-time Outbreak and Disease Surveillance) [11], and GEIS (Global Emerging Infections Surveillance and Reporting System) [12]. A detailed overview of these systems and their applications is further described in Shmueli and Burkhom [13]. These systems include tools for natural language processing (NLP) for parsing unstructured textual data, basic statistical analyses tools, time-series counts/ratios as well as geographic information system (GIS) based visualization that can summarize to the end-user the nature or urgency of an emerging infectious disease. It must be noted that most tools developed are specific to infectious diseases; although the tools can be used to analyze other non-infectious diseases, they are very rarely utilized for monitoring such conditions.

Other public health monitoring systems such as Google Flu Trends [14] utilize internet search patterns of users to predict the incidence of flu at local, regional (state-wide) and national levels. While initial studies have shown that tools that make use of "proxy" datasets can serve as useful monitors for emerging diseases [14,8,17], recent studies have demonstrated that the estimates from internet search patterns can over-estimate the severity of the outbreak [18,19]. Self-reporting tools such as micro-blogging and social media are also becoming effective proxies for public health surveillance [20-24], although such datasets also have relatively higher noise and teasing out relevant information for specific disease conditions can be quite challenging [25].

In recent years, the availability of electronic health records (EHR) [26,27] and electronic healthcare reimbursement claims (or briefly, claims) [28-30] have proven to be valuable resources for collecting, monitoring and analyzing public health-related questions. While EHRs refer to an individual patient's medical history collected and processed at individual medical facilities (such as a clinic or hospital), claims refer to electronic records of claim transactions processed by retail pharmacies (and/or clinics). EHR and claims provide rich and timely information regarding prevailing medical conditions in any given geographic area; however, the use of EHR and claims for public health surveillance is still in its early stages. Privacy and security-related concerns, data disparity across diverse/individual clinics/hospitals, as well the sheer complexity involved in aggregating and processing such large-scale structured datasets can pose significant data analytic challenges for even simple public health surveillance tasks [31,32].

Thus, within the context of public health surveillance, the availability of these diverse datasets pose two immediate 'big data' challenges: (1) scalable, seamless and uniform access to diverse datasets and (2) scalable data analytic techniques that can provide rich feedback to the end-user regarding emerging public health emergencies [33]. While there is significant agreement within the public health community regarding the use of data analytics and informatics techniques as being central to the success of any biosurveillance program, the development of machine learning and data analytic techniques specifically designed to handle heterogeneous datasets at massive scales has been especially challenging. These challenges can be attributed to the lack of standards and tools that facilitate data/information exchange and secondly, to the lack of available data analytic frameworks that can automatically integrate heterogeneous datasets and analyze them in near real-time to provide insights into emerging public health problems. Additionally, the complex etiologies of diseases pose special challenges in developing analytic tools to monitor them. For example, the symptoms of the common flu and a serious outbreak such as West Nile virus can be very similar, but teasing out these symptoms from a context specific search of Twitter and other social media data can be quite challenging. Therefore, there is a need to develop novel machine learning tools that can not only handle large datasets, but can also simultaneously examine heterogeneous data sets to identify emerging patterns of disease spread across geographically distributed regions.

### Oak Ridge biosurveillance toolkit for public health surveillance and dynamics

In this paper, we outline our recent efforts in developing novel machine learning tools for public health surveillance addressing the aforementioned big data challenges [34]. The Oak Ridge Biosurveillance Toolkit (ORBiT) is being developed as a machine learning platform that processes both direct and indirect data sources by integrating insights from heterogeneous datasets for answering public health surveillance-related queries. In contrast to existing systems where the primary emphasis is on data collection, archival and visualization of specific datasets, ORBiT is being developed as a distributed, component based platform for novel statistical and machine learning tools that can provide insights into spatial and temporal patterns of public health emergencies. By tightly integrating the machine learning tools with visual analytics interfaces in a web-based framework, ORBiT allows analysts and other end-users to explore heterogeneous datasets to detect patterns/correlations across different data streams, identify emerging disease outbreaks and forecast outbreaks and monitor control strategies.

We illustrate the applicability of ORBiT to identify, quantify and describe spatial and temporal patterns of the 2009-2010 pandemic H1N1 flu within the United States (US) from an infectious disease surveillance perspective. We illustrate how the integration of heterogeneous data sources, including publicly accessible data from the US Centers for Disease Control (CDC), openly accessible data from Google Flu Trends and claims obtained from a private organization that consolidates diagnostic and prescription electronic transactions can provide timely and novel information regarding how the 2009-2010 influenza pandemic affected the entire US. Our analysis of these datasets shows that a small number of distinct temporal patterns govern how the pandemic spread throughout the country. Additionally, we extract intrinsic multi-scale patterns from the claims data, moving successively from local to regional to national patterns. These patterns depict the process by which the H1N1 flu spread across the entire country in distinct waves, each with its own unique temporal and spatial signatures. Although this study is a retrospective analysis of the 2009-2010 flu season, we show that the patterns can also translate into meaningful insights for future years, especially to interpret baselines. Taken together, our study provides a summary of ORBiT capabilities and how it can be used as a scalable platform for public health surveillance.

## Methods

In this section, we outline how ORBiT can incorporate claims data to discover spatial and temporal patterns from the 2009-2010 pandemic H1N1 flu season. The description of the ORBiT framework is provided elsewhere [34]. In this paper, we describe the claims data and the use of non-negative matrix factorization (NMF) as a novel technique to analyze claims data to automatically discover spatial and temporal patterns.

### Dataset description

#### Electronic healthcare claims reimbursement data from IMS Health

IMS Health is a leading consolidator of claims within the US, collecting over 55-60 million claims every week. This proprietary dataset therefore constitutes a unique resource for public health surveillance. Two types of claims are collected by IMS Health: (1) diagnostic data (referred to as claims) which processes claims from over a million medical practitioners/physicians every year received from all parts of the US, including urban and rural areas; (2) prescription data (referred to as Rx), which processes prescription claims from retail pharmacies within the US. The claims data consists of over 1 billion claims collected annually and represents over 165 million unique patients. The Rx data consists of over 3 billion claims collected annually and provides for a rich resource to monitor and track drug delivery and efficacy across the entire country. IMS Health uses proprietary technology to protect patient privacy and all of the data available/used for analysis are HIPAA-compliant.

For this study, we analyzed the IMS Health claims data from Apr 1, 2009 - Mar 31, 2010, with a total of nearly one billion records. The claims data was processed for flu-related records using the definition shown below:

**• Flu case definition: **include only hospital diagnosed cases of the flu, namely ICD9 codes 486XX and 488XX.

The definition of the flu corresponds to hospital diagnosed cases of the flu, which provides a specific count in terms of the number of flu cases recorded within any zip code. The reason we focus on this stricter definition is to count only cases that we know would have been diagnosed with the flu and exclude other symptoms that perhaps can bias the observations based on generic symptoms such as sore-throat, cough and fever. For organizing the data based on a specific geographic location, we used the provider's primary five digit zip code that was directly accessible from the claims data. Note that this assumption is reasonable, given that the patient's service provider/pharmacy is most likely to be co-located unless the patient remotely consults with his/her provider. In the current study, only 0.0001% of the total records showed different 3 digit zip codes for the patient and their service provider.

The claims data is usually reported every day with claims coming into the data warehouse continuously. However, due to claims submission delays by healthcare providers and internal data-processing and cleaning, there is a lag between the service date (i.e., the date on which the physician issued the diagnosis) versus the date on which the data was actually loaded/processed with the IMS Health data warehouses. Since the spatial resolution of the claims data is at the zip-code level, we defined local metropolitan areas (for cities) and the different geographic regions (see below) based on an aggregation of data from these individual zip codes, thus maintaining consistency between the definitions of individual zip codes all the way to the entire nation.

#### CDC Influenza like Illnesses Network (ILINet) data

The US CDC maintains information on patient visits to health care providers for influenza like illnesses (ILI), which consists of more than 2,900 outpatient healthcare providers with the ability to track more than 30 million patient visits every year [35]. The data reported every week consists of the total number of patient visits as well as the total number of patients with ILI-like symptoms organized by age groups. ILI cases are defined based on observing fever (temperature of 100°F or 37.8°C or greater) and a cough/sore throat without a known cause other than influenza. The CDC then baselines these reports based on the state population and defines several metrics for individual geographic regions. These regions, known as the Health and Human Services (HHS) regions are summarized in Table 1. Although there are different forms of ILI surveillance including influenza-associated pediatric mortality surveillance and influenza hospitalization network (FluSurvNet), for this current study, we used the publicly available ILINet data [36].

**Table 1:** Summary of coverage in IMS claims data.

Region	States	**Z***_GIS_*	**Z***_claims_*	% coverage
HHS-I	CT, ME, MA, NH, RI, VT	1781	813	45.7
HHS-II	NJ, NY	2279	1242	54.5
HHS-III	DE, DC, MD, PA, VA, WV	4019	1623	40.4
HHS-IV	AL, FL, GA, KY, MS, NC, SC, TN	5470	2836	51.8
HHS-V	IL, IN, MI, MN, OH, WI	6012	2647	44.0
HHS-VI	AR, LA, NM, OK, TX	4134	1744	42.2
HHS-VII	IA, KS, MO, NE	3315	996	30.0
HHS-VIII	CO, MT, ND, SD, UT, WY	2105	597	28.4
HHS-IX	AZ, CA, HI, NV	2382	1391	58.4
HHS-X	AK, ID, OR, WA	1551	585	37.7

#### Google Flu Trends data

The Google Flu Trends (GFT) project [14] builds an automated method for discovering influenza-related search queries by aggregating historical logs of online web search queries and developing a log-linear model that estimates the probability that a query is related to ILI. This model was validated across CDC-observed ILI percentages and made available for the public from http://www.google.org/flutrends/us/#US. We downloaded the weekly information available for the same period covered by the IMS Health claims data (Apr 1, 2009 - Mar 31, 2010).

### Using Non-negative matrix factorization (NMF) to extract spatial and temporal patterns from claims data

One of the many advantages of using claims data for public health surveillance is that it provides information about ILI-incidence at individual zip code level resolution. Unlike the ILINet data, which statistically aggregates total counts of ILI-symptoms over the entire US from vast geographic regions, the claims data can be used to obtain fine-grained details about specific regional variations and how that may have impacted the quick spread of the 2009-2010 pandemic flu throughout the US. To explore the further use of claims data and to perform a retrospective analysis of the 2009-2010 pandemic flu within the US, we organized the ILI-related data from claims into a matrix A, that has the overall dimensions of *N_z _*× *N_t_*, where *N_z _*represents the total number of zip codes and *N_t _*represents the total number of time points (365 days).

Based on the comparison of the ILINet and GFT data presented above, we *hypothesize that the flu incidence patterns are categorical in space and time*. This is reasonable, especially given the geographic vastness of the US, the spatial (individual zip codes) and temporal (daily reports of ILI-conditions) resolution of the claims data. Given prior knowledge that there are at least three distinct 'peaks' associated with the 2009-2010 pandemic [37], we want to extract low-dimensional representations for this claims data. Further, the flu incidence matrices have non-negative entries (i.e., it is not possible to obtain a negative count of patients reporting flu symptoms at a zip code). Hence, we used non-negative matrix factorization (NMF) as a technique to extract low-rank approximations from the claims data.

Given a data matrix **A **with non-negative values, with dimensions *N_z _*× *N_t_*, NMF finds low-rank approximation (*s*) of the form **A = WH**, where **W **(*N_z _*× *s*) captures spatial patterns and **H **(*s *× *N_t_*) describes temporal patterns within the data. Using the alternate least squares algorithm proposed by Paatero, available as part of Matlab, we ran NMF for 1,000 iterations. To identify an appropriate low-rank subspace (*s*), we iterated over *s *= 1 . . . 15, dividing the original data into training and testing data. We tracked the residual errors using the Frobenius norm for both training and testing data. For each choice of *s*, we performed a total of 250 iterations. Once the optimal s was selected, we report the most stable version of the basis matrices (**W, H**) by computing the KL divergence between every pair of the 250 instances of **W **from the training set and picking **W **with the lowest KL divergence value.

## Results

### Influenza like Illnesses (ILI)-related claims data provide higher spatial and temporal resolution into ILI-case counts within the US

One of the primary goals for our study was to quantitatively assess the timeliness and coverage (both in space and time) of the IMS Health claims data. As part of this exploratory study, we extracted the data as described in the Methods section and compared this data with CDC ILINet and GFT datasets. Note that both CDC ILINet and GFT data are known to be correlated [14,18], however both datasets correspond to different modalities. While the CDC ILINet data is primarily focused on out-patient visits, GFT uses search patterns of users to identify patterns of influenza occurrence. In spite of the differences in data collection and curation, we hypothesize that the IMS Health claims data, based on the case definition used in this current study will closely match the temporal trends observed from the CDC ILINet and GFT data.

#### ILI-incidence in claims are consistent with ILINet and GFT data across HHS regions

**Figure 1:**
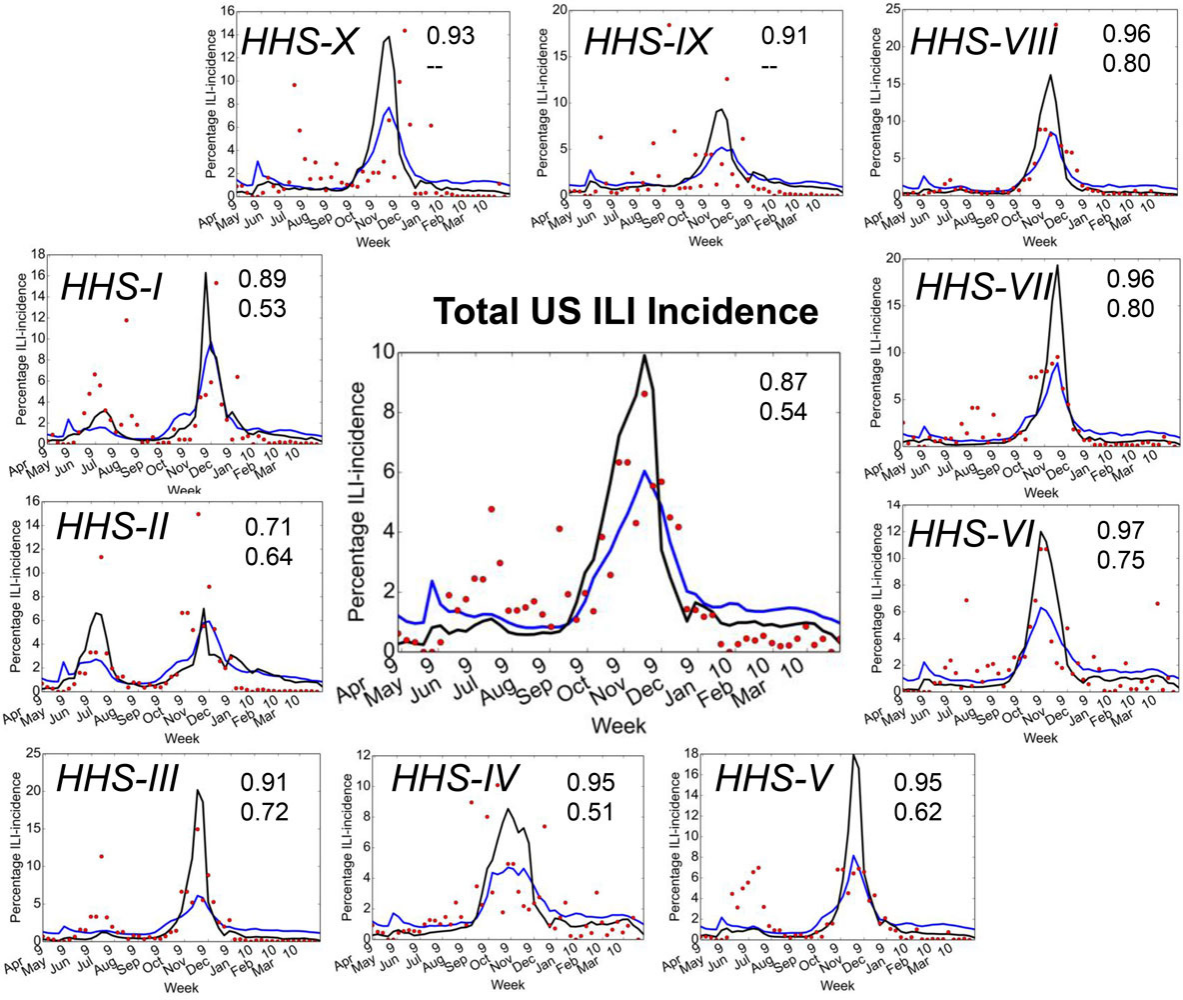
**Temporal trends of ILI incidence from IMS Health claims, CDC ILINet and Google Flu Trends (GFT) during the 2009-2010 pandemic flu show significant similarities**. The total incidence of H1N1 pandemic as provided by GFT (blue line) and CDC (red dots) are plotted together with IMS claims data (black line). Note here that we used the strict definition of the flu (ICD9 codes: 486XX and 488XX). The temporal trends for the entire US are plotted in the center, followed by the 10 Human and Health Services (HHS) Regions shown around the US (HHS-I to HHS-X). In all the cases, the agreement between IMS claims data, GFT and CDC ILINet data is quantified by the correlation coefficient, depicted on the side of each panel. The numbers at the right hand side of every panel represent the correlation coefficient between the IMS claims and GFT data (top) and the IMS claims with ILINet data (bottom) respectively. These numbers represent all the data from the 52 weeks collected instead of measuring across the time segments for which CDC ILINet data was available. Note that CDC ILINet data has some missing values, removing these segments from our analysis actually improves the correlations (see Main Text for discussion). For HHS-IX and HHS-X, the CDC ILINet data was not fully available at the time of download and hence we have not shown the correlation values.

We compared the ILI-incidence data at two spatial scales: (1) the overall country (Figure 1 center panel) and the ten HHS regions (Figure 1 HHS-I through HHS-X panels). To ensure that we were comparing similar quantities, we converted the counts from the ILI-incidence rates from claims data into percentages, in a similar way outlined in previous papers. Not surprisingly, the overall US ILI-incidence rates over time reflects a similar behavior across both the claims and GFT data. The average agreement (quantified by the Pearson correlation) between the GFT and claims data is about 0.9 (with a p-value of 4E-11), even within individual HHS regions. However, the similarity is less pronounced with respect to the agreement between the CDC ILINet data and IMS Health claims data; we speculate that the publicly available information from ILINet has several incomplete entries for the same time period. Therefore, when we compare the data in a similar way to the GFT approach by removing the missing entries and extracting time segments which have reported data available, the agreement increases to about 0.9 (p-value of 4E-11).

Within the entire nation, the flu incidence peaked around the time of Oct-Nov 2009, which is reflected in all the three data streams examined. The percentage ILI-incidence is significantly less within CDC ILINet and GFT. We believe that this may be a consequence of the nature of data collection techniques used in each case. GFT data relies on a statistical model to identify search queries related to influenza. The ILINet data is primarily collected from outpatient visits and covers a small portion of the primary care facilities within the entire country. The claims data is, however, dependent on the primary care physician's reporting of transactions, which can vary across the nation (see next subsection). Furthermore, the reported number of cases within the claims data can be regarded as upper-bound estimates of the true infection (since not all diagnosed cases of the flu are true positives).

Even across different HHS regions, we observe that the claims data consistently presents higher number of ILI cases compared to the CDC ILInet and GFT. In particular, we note that except for HHS-I and HHS-II, which show the presence of two distinct peaks in ILI- incidence (reflected in all the three data streams), all the other regions consistently show that the peak of the pandemic occurred around the Oct-Nov 2009 time-frame. As is well documented from previous studies [38,37,39], HHS-I and II correspond to the northeast (states including NY, NJ, CT, ME, MA, NH, RI, VT) that exhibited a distinct early onset of the flu pandemic followed by the peak observed in Oct-Nov 2009. Although we observe that the ILINet data shows the presence of early onset within HHS-III as well as HHS- V, both claims and GFT do not show such a pronounced outbreak in these regions. The data from ILINet does not fully cover the time-span examined, especially since there are a number of weeks with missing data. Excluding those missing time-segments across the entire nation, the overall Pearson correlation between ILINet and claims data is about 0.86 (p-value = 5.43E-11) indicating that there is significant similarity between the two datasets.

The coverage of the claims data is not uniform throughout the country. Although within the different HHS regions the total ILI-incidence rates are higher on average within the claims data, the coverage of ILI incidence within individual HHS regions can vary. As summarized in Table 1 the total number of zip codes within each HHS region for which claims data is available varies from a minimum of 28.4% to a maximum of 58.4%. The reporting from the claims data is fairly consistent across these regions for any given year, as reflected by the total number of diagnostic records available at these regions. Through these observations, we can conclude that the claims data provides similar insights into public health surveillance as traditional sources such as CDC ILINet.

### Non-negative Matrix Factorization (NMF) identifies distinct spatial and temporal patterns from the 2009-2010 pandemic H1N1 flu season

**Figure 2:**
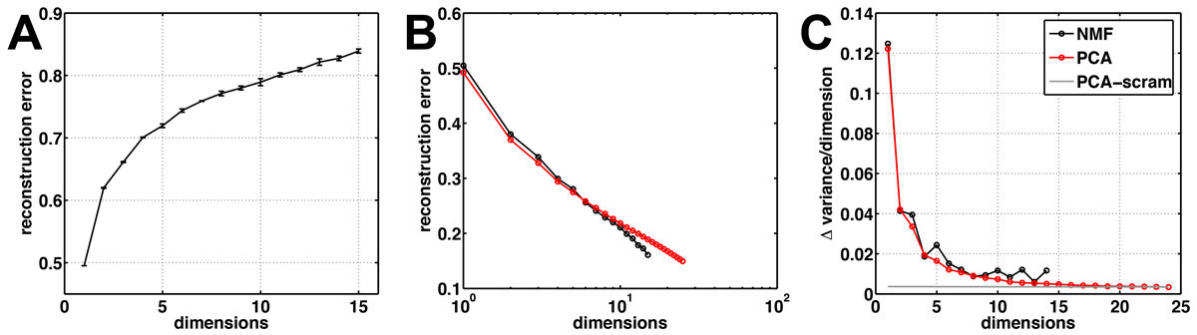
**Summary of non-negative matrix factorization (NMF) applied to ILI diagnostic claims claims data**. (A) Reconstruction error or the fraction of unexplained variance for PCA (red) and NMF (black) versus the subspace *s *selected. (B) Change in reconstruction error for PCA and NMF as compared to the change in reconstruction error for PCA performed on a scrambled version of the input matrix **A**. PCA_scram _shown in gray line is used to estimate the cut-off number of dimensions, beyond which the dimensionality reduction method explains only noise within the dataset. For our analysis, s beyond 12 is only explaining noise in the data, as is evident from the intersection between the gray and black/red lines.

We defined a zip code as having statistically significant data if it reported at least 10 cases of the flu in any given week of the year. This simple threshold based filtering allowed us to remove any zip codes that had very few cases reported throughout the year. Based on this simple filtering, the total number of zip codes with reported flu cases (*N_z _*) was 14,098 and we used *N_t _*to be 365 days. Instead of examining weekly reports as discussed above, we used a daily resolution to fully leverage the claims data. Further, we also wanted to test the hypothesis that a daily resolution of the pandemic flu season will provide fine-grained insights into distinct patterns of how the flu spread. As summarized in Figure 2, only a small number of dimensions are sufficient to describe the pandemic flu outbreak throughout the US. To select the number of dimensions, we plotted the reconstruction error (i.e., fraction of unexplained variance) versus the subspace for the 250 repetitions of NMF (Figure 2A), and compared this with the reconstruction error obtained with PCA performed on the original data (PCA_orig_) and the scrambled data (PCA_scram_; Figure 2B). As observed, the slope of PCA_scram _is quite small and relatively constant for increasing subspace sizes. This provides a means to estimate the subspace size beyond which a given model is explaining noise rather than correlations in the data [40]. To visualize this cut-off, in Figure 2C we plot the change in variance for each added dimension (differences between successive points in Figure 2B). The reconstruction error rates of both PCA_orig _with PCA_scram _at subspace around *s *= 12. Although it is possible to choose *s *= 12 and describe the spatial and temporal patterns, we use a smaller subspace (*s *= 5) to describe the 2009 H1N1 pan- demic. This is mainly due to the fact that we wanted a simpler representation of this high dimensional space and traded the interpretability of a lower dimensional representation for the complexity of patterns when *s *= 12. Further, lower number of dimensions (*s *< 5) do not provide a clear separation of the temporal/spatial patterns and hence we have chosen to detail our analysis with a subspace size of 5.

#### NMF identifies multi-scale ILI-breakout patterns within the US

**Figure 3:**
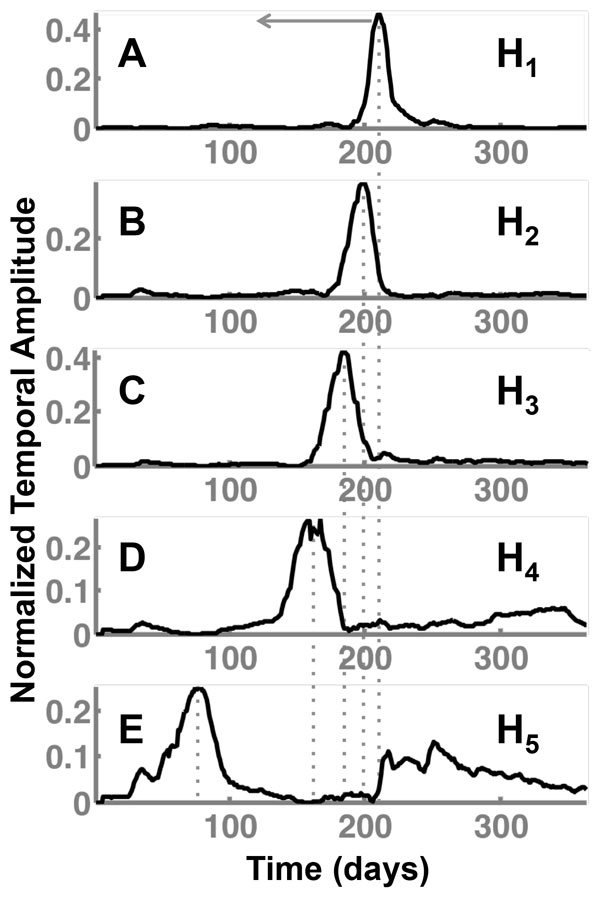
**Five distinct temporal patterns govern how the pandemic flu spread throughout the US**. The normalized temporal amplitude is plotted against the total number of days (Apr 1, 2009-Mar 31, 2010). Observe the distinct lag in each of the five patterns, with successive **H***_i _*indicating the peak shift occurring towards the left (indicted by a gray arrow). These patterns summarize the different peaks during the H1N1 pandemic. Notably, H_1_, H_4 _and H_5 _capture the late, middle and early H1N1 pandemic peaks occurring within the entire country.

A summary of the five temporal patterns as extracted from NMF using **H **is depicted in Figure 3. There are distinct peaks for each of the five patterns, indicating a unique phase for the 2009-2010 pandemic flu. Interestingly, the peak of ILI-incidence across each of the temporal patterns is left shifted - indicating a lag period in the ILI incidence rates observed across the different geographic regions (see next subsection). Notably, **H**_1 _shows a peak in ILI incidence around day 206-210, corresponding to a time period of Oct 24, 2009; **H**_2 _shows peak about a week earlier (Oct 18, 2009) followed by **H**_3 _peaking around Sep 28, 2009 and **H**_4 _showing a peak of Aug 19, 2009. **H**_5 _corresponds to an early flu outbreak observed in the early-middle spring time (May 31-Jun 5, 2009), which was distinctly observed across the North east HHS regions in Figure 1. Another notable aspect of **H**_5 _is that the early peak of the ILI-incidence is followed by a secondary peak in and around the same time of **H**_1_, indicating that the likely presence of an early flu season (in the spring season) also influenced the late peaks observed in the fall season (see below for explanation of the spatial patterns observed). Thus, these ILI-breakout patterns provide a succinct summary of how the 2009-2010 flu season affected the entire country.

**Figure 4:**
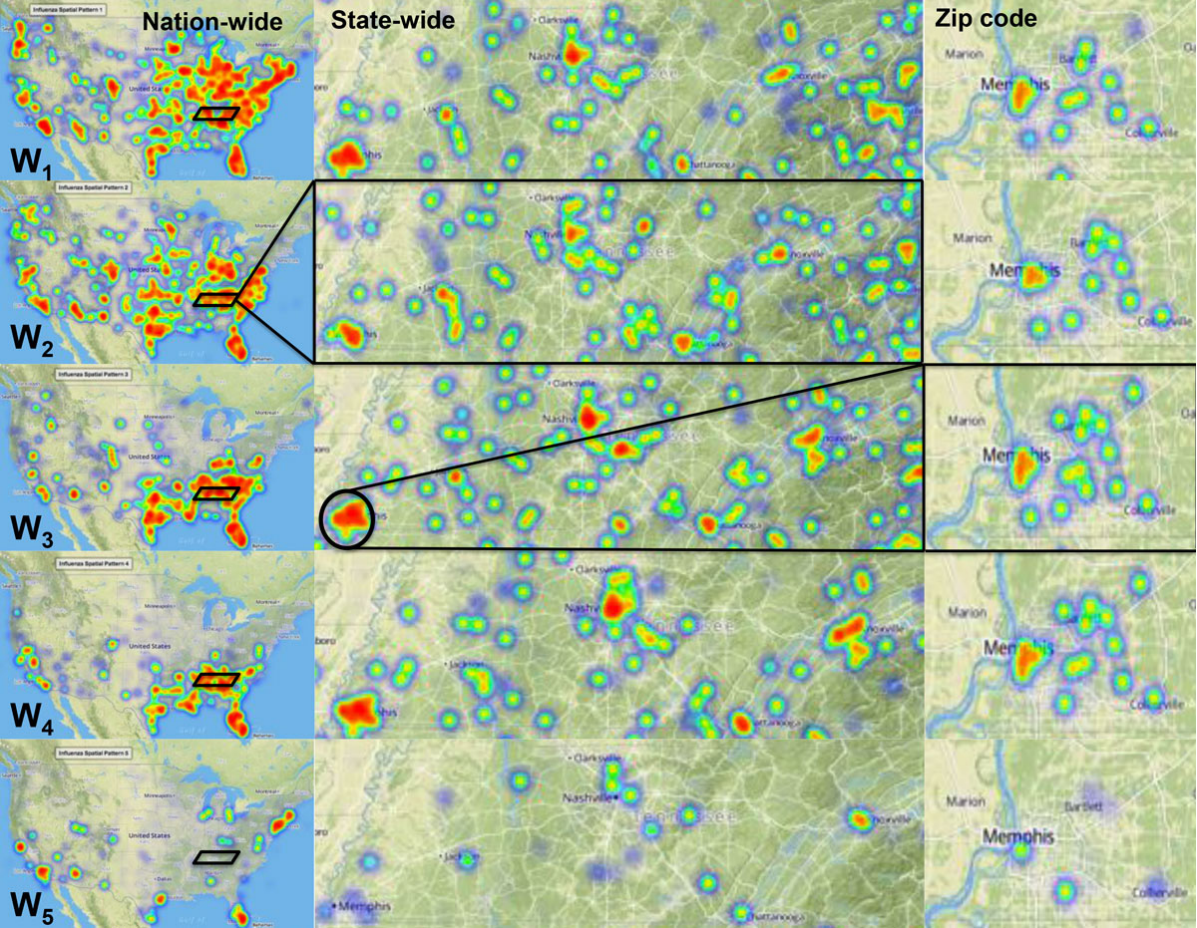
**Multi-scale spatial patterns of H1N1 influenza occurrence in the US**. Each of the spatial pattern W discovered from NMF can examine how the flu spread throughout the US (left hand panels). The nation wide panels depict how **W**_1 _pattern is widespread throughout the US followed by progressively moving down south (**W**_4 _). The spatial pattern **W**_5 _depicts flu prevalence only within large metropolitan areas and southern Florida. One can focus further into state-wide patterns (middle panel) and examine how ILI-patterns affect the state of Tennessee and towards specific metropolitan areas (e.g., Memphis in Tennessee, right most panels) and capture minor variations in the ILI-patterns according to different zip-codes. These differences also allow one to identify bridge regions (highlighted by red and magenta circles) that show more than two ILI-patterns in the same zip code. These analyses can be further extended out towards the state and nation-wide areas.

As shown in Figure 4, each of the **W***_i _*vectors provides a specific spatial (geographic) pattern during the 2009-2010 pandemic flu season. The advantage of this representation is that NMF allows us to interpret and visualize the pandemic flu season as a multi-scale spatial model that captures nation-wide, state-wide and zip code specific behaviors observed during the pandemic flu season. Specifically, each **W***_i _*depicts how the flu encompassed the entire nation. The matrix representation of **W **provides a succinct summary of the flu prevalence across the individual zip codes, which can be visualized on a geographic map of the US shown in Figure 4 (labeled National). In this map, darker colors of red correspond to a higher flu prevalence in the region, whereas lighter colors (orange, yellow, green and blue) represent a lower flu prevalence pertaining to a specific spatial pattern.

One of the notable observations from our analysis is that the flu prevalence patterns reveal distinct areas were affected by each **W***_i_*. For example, while **W**_1 _occurs throughout the US encompassing both the northeast and northwest regions of the country, **W**_4 _is primarily observed in the southeast and western regions (California) of the country. The pattern **W**_5 _is exclusively observed within larger metropolitan areas (large cities with at least 1 million people during the 2010 census period). It is also interesting to point out that all the five patterns are observed within metropolitan areas, perhaps reflecting the dynamics of people moving between these large cities. (It is also important to point out here that additional data would be required to validate this observation, which we are not pursuing as part of this paper.)

At the state level, we can describe how the flu patterns uniquely affected different counties/regions, as shown in Figure 4 (State-wide panel). Here we have highlighted the state of Tennessee (TN; for which the coverage of the claims data was about 47%). As a south-eastern state, TN was widely affected by the H1N1 pandemic. While the major cities of TN including Memphis, Nashville, Knoxville and Chattanooga - all exhibit the five patterns, the individual county areas around the major cities have unique spatial patterns within each **W***_i_*, depicting that the flu prevalence pattern was indeed unique to different areas within the state (as highlighted by the yellow rectangles in the figure). Such an argument can also be extended to the city/zip code resolution (right most panel), whereby each pattern captures how neighborhoods or suburb regions show unique prevalence patterns as one navigates the different spatial patterns from **W**_1 _to **W**_5_.

#### Identifying "bridge regions" within break-out patterns in the US

It is interesting to note that between the different **W***_i _*a small subset of the zip codes act as *bridge regions*. We define these bridge regions to be zip codes that exhibit more than one **W***_i _*at the same time-period. These zip codes, not surprisingly, are concentrated towards the different suburb regions of the different metropolitan cities in the state. For example, in the city of Memphis, there are distinct bridge regions where we observe that a cluster of three zip codes in the area corresponding to Bartlett (highlighted by a red rounded rectangle in Figure 4 across all the three spatial patterns) that exhibit **W**_2_, **W**_3 _and **W**_4 _patterns. Notably, this region showed very little flu during the early half of the season. Another example of a bridge region is highlighted by a purple circle in Figure 4 where **W**_1_, **W**_3 _and **W**_4 _patterns dominated in the suburbs of East Memphis. Only the area of Bartlett and Collierville show the presence of the early and late fly patterns (corresponding with the spatial pattern **W**_5_).

**Figure 5:**
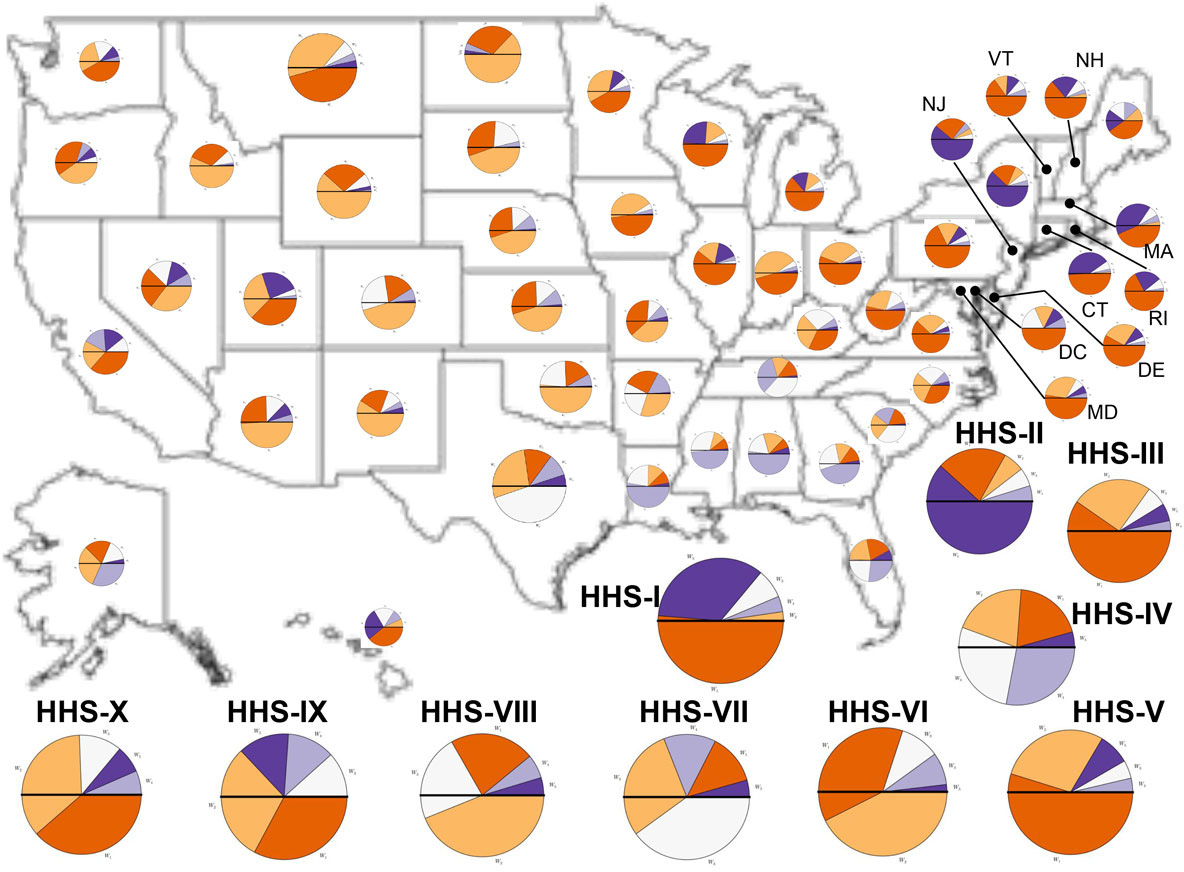
**A small number of regions within the US act as bridge regions for the 2009-2010 H1N1-flu season**. Within every state, we quantify the extent to which the individual spatial patterns are dominant using a pie-chart representation. The colors represent respective spatial patterns (W_1...5_), as highlighted in the legend. In the pie-chart, a line in the middle points out the 50% cut-off for a particular flu pattern and is used as a guide to identify dominant patterns. For the individual HHS regions shown below, we can see a dominant pattern, within the individual states, (for e.g., MA, CT, MT, CO, MS) more than one pattern dominates indicating the complexity of how the H1N1 flu spread within these regions. Note that the patterns also correspond to the time when the flu peaked in these individual regions and hence such patterns are instructive in visually interpreting how the different spread patterns affected an individual state.

Based on this initial analysis, we can identify bridge regions at the state-and national-level by aggregating the spatial patterns to the respective scales. Instead of examining specific spatial patterns, we examine the most dominant spatial pattern (**W***_i_*) in a given state or HHS region. A dominant pattern is defined as a spatial pattern that is prevalent in a specific zip code based on the maximum **W***_i _*value(s) within the zip codes that constitute the state (or HHS region). For this study, we decided to use a simple threshold of 50% to determine if a spatial pattern was dominant in that state/region. As summarized in Figure 5, the individual pie charts within each state captures the percentage contribution of each **W***_i _*that was dominant in that region, which provides an intuitive visual analysis of the regions impacted by the 2009 H1N1 pandemic.

For the different states, one can identify the most dominant pattern just by examining how prevalent these flu patterns were across the different zip codes across a particular state. While states like Wyoming, North Dakota, Pennsylvania and others show a dominant, single spatial pattern, states such as Georgia, California, Nevada and Tennessee exhibit typically two patterns that dominate these regions. Thus, states such as Kentucky and Tennessee act as bridge regions in the spread of the pandemic.

Extending this analysis further for each of the HHS regions, we observe that HHS-IV and HHS-VIII are dominated by two patterns (completely different in these regions), where as other HHS regions including HHS-I-III and HHS-V-VII have a single dominant spatial pattern that is prevalent in at least 50% of the zip codes in these regions. Interestingly, the entire southeast acts as a bridge region showing the presence of two or more patterns simultaneously occurring within 50% of the zip codes. Similar observations can be made also within HHS-IX and HHS-X, where **W**_1 _and **W**_2 _dominate. We also observe that the northeast part of the country exhibits only **W**_1 _and **W**_5_, confirming further that the early flu peaks were prevalent only in these regions (apart from other major metropolitan areas). It is also interesting to note that the very same regions that show **W**_5 _also exhibit a temporal coupling between the early and late part of the flu. These regions, especially in the northeast (HHS-II) were affected by an early peak of the H1N1 pandemic followed by a sustained incidence of the flu even after the entire nation had more or less recovered from the major outbreaks.

## Discussion and conclusion

In this paper, we examined the use of the diagnostic data to reveal spatial and temporal patterns of how the 2009 H1N1 pandemic affected the entire country. To our knowledge, the use of NMF in the context of extracting spatio-temporal patterns of disease spread is novel and the break out patterns extracted from the claims data provide specific insights into the 2009 pandemic. The break out patterns show how different parts of the US were vulnerable and highlight regions that may have needed additional attention as the pandemic was spreading through the nation. The patterns also describe the multi-scale nature of flu outbreaks beginning with the individual zip code resolution all the way to the entire nation, capturing the complex dependencies that may have had an impact in spreading the pandemic. Our analysis also reveals specific features of the flu outbreak patterns that highlight the differences between both urban (metropolitan) and rural areas. The patterns extracted are categorical in that they describe the overall dynamics of the pandemic in distinct phases through out the nation. While the patterns have intuitive interpretative power, more quantitative measures of the distinct spatial and temporal coupling patterns are required.

At this time, because we have not integrated socio-economic/census data into our analysis, it is difficult for us to speculate whether particular demographic factors (e.g., age-group, socio-economic background or other factors), population density or other environmental and climatic factors within these regions lead to the observed patterns. We also note the relatively sparse coverage of the claims data across the country and these regions also constitute large parts of the US where the population density is quite low. A more systematic analysis of the variation in population of these regions, followed by a statistical comparison with the flu diagnostic data would be necessary to draw additional conclusions regarding the epidemiological significance of these spatial and temporal patterns.

Although in this paper, we do not describe the many confounding factors (e.g., environmental factors/ climate factors that have a strong influence on the occurrence of asthma) that may play a role in the co-occurrence of asthma and flu, the ability to discover such complex associations from claims provides an added capability for public health surveillance systems to monitor and quickly identify vulnerable geographic areas/population for preemptive intervention. We must note here that a more detailed analysis of the spatio-temporal patterns is required. Additionally, within the scope of this paper, we have not examined whether these patterns correspond to other well known algorithms such as Google Flu. Finally, we must also note that the predictive aspects of our algorithm have also not been fully explored for two reasons: (1) the data available to us is only from the 2009-2010 flu season and (2) it is difficult to obtain a baseline behavior based on a year that showed highly anomalous behavior in terms of the overall flu incidence across the entire country. We will explore these questions in greater detail in a following publication.

While diagnostic information (from claims data) can be helpful for public health surveillance, additional analyses of the prescription datasets (Rx) from IMS Health is necessary to obtain precise insights regarding the pandemic spread. The prescription transactions, in addition to providing counts of patients that were prescribed anti-viral medications, also record the dosage of these drugs and hence can provide tighter bounds on the number of estimated people infected and measure the intensity of spread. Such a collective integration of claims and Rx datasets can provide novel insights not only in the context of understanding the flu, but can have a wide impact in general for more complex disease etiologies and chronic disease conditions.

### Incorporating H1N1 molecular evolutionary information into ORBiT

The spatial and temporal patterns discovered from the claims data and NMF can be considered as approximate representations of epidemiological curves obtained from traditional disease spread (either compartmental or agent-based) models. The temporal patterns shown in Figure 3 indicate different phases of the H1N1 flu epidemic. The multi-scale representation of the H1N1 epidemiological spread can be used as starting points for other complex types of analysis. For example, one extension would be to include evolutionary history of different H1N1 viral strains. The recent availability of large-scale sequence databases such as GISAID [41] can provide insights into specific viral strains that are prevalent within a geographic region. Tracing the phylogenetic relationship between different strains of the virus, we can then estimate parameters for disease spread models [42]. We can also incorporate the evolutionary information into statistical models [43,30] to understand the how viral evolution affects the disease spread process. Further, these patterns can be examined to identify regions that are vulnerable to specific strains and target them for early intervention. Such enhancements will be evaluated in forthcoming publications from our group.

### Other capabilities within ORBiT

ORBiT is designed as a toolbox for developing machine learning tools that can aid public health surveillance. Within the scope of this paper, we have demonstrated the use of novel diagnostic (claims) datasets to discover a small set of spatial and temporal patterns that characterize the 2009-2010 pandemic H1N1 flu. However, we have not described all the capabilities within ORBiT. Apart from supporting machine learning algorithms from direct sources for public health surveillance, ORBiT can be used in other contexts including (1) extracting and analyzing emerging, indirect datasets for public health surveillance, e.g., Twitter [34]; and (2) integrating datasets such as claims to estimate parameters for disease spread models so that one can turn the analytical power from the aforementioned application into predictive models that can aid decision makers with more accurate insights [44]. We hope to examine these applications in greater detail in future studies.

## Competing interests

The authors declare that they have no competing interests.

## Authors' contributions

AR and LP conceived and designed the study. SV provided the data. TH and LP processed and stored the eHRC datasets for analysis. AR, LP, SQ, and CC developed the analysis techniques. TH and CAS developed the user interface components for visualizing the results. AR, CC, SQ, and TH analyzed the data. AR, LP, TH, CAS, SQ, SV, and CC wrote the paper.

## Declarations

Publication of this article was funded by ORNL SEED funding to LP.

Oak Ridge National Laboratory (ORNL) is operated by UT-Battelle, LLC, for the U.S. Department of Energy under contract DE-AC05-00OR22725. The United States Government retains and the publisher, by accepting the article for publication, acknowledges that the United States Government retains a non-exclusive, paid-up, irrevocable, world-wide license to publish or reproduce the published form of this manuscript, or allow others to do so, for United States Government purposes.

This article has been published as part of *BMC Bioinformatics *Volume 16 Supplement 17, 2015: Selected articles from the Fourth IEEE International Conference on Computational Advances in Bio and medical Sciences (ICCABS 2014): Bioinformatics. The full contents of the supplement are available online at http://www.biomedcentral.com/bmcbioinformatics/supplements/16/S17.
